# Role of Vitamin E and the Orexin System in Neuroprotection

**DOI:** 10.3390/brainsci11081098

**Published:** 2021-08-20

**Authors:** Maria Ester La Torre, Ines Villano, Marcellino Monda, Antonietta Messina, Giuseppe Cibelli, Anna Valenzano, Daniela Pisanelli, Maria Antonietta Panaro, Nicola Tartaglia, Antonio Ambrosi, Marco Carotenuto, Vincenzo Monda, Giovanni Messina, Chiara Porro

**Affiliations:** 1 Department of Clinical and Experimental Medicine, University of Foggia, 71122 Foggia, Italy; ester.latorre@unifg.it (M.E.L.T.); giuseppe.cibelli@unifg.it (G.C.); anna.valenzano@unifg.it (A.V.); daniela.pisanelli82@gmail.com (D.P.); chiara.porro@unifg.it (C.P.); 2 Department of Experimental Medicine, Section of Human Physiology and Unit of Dietetics and Sports Medicine, Università degli Studi della Campania “Luigi Vanvitelli”, 80100 Naples, Italy; ines.villano@unicampania.it (I.V.); marcellino.monda@unicampania.it (M.M.); antonietta.messina@unicampania.it (A.M.); vincenzo.monda@unicampania.it (V.M.); 3 Department of Biosciences, Biotechnologies and Biopharmaceutics, University of Bari, 70125 Bari, Italy; mariantonietta.panaro@uniba.it; 4 Department of Medical and Surgical Sciences, University of Foggia, Viale Pinto, 71122 Foggia, Italy; nicola.tartaglia@unifg.it (N.T.); antonio.ambrosi@unifg.it (A.A.); 5 Clinic of Child and Adolescent Neuropsychiatry, Department of Mental Health, Physical and Preventive Medicine, Università degli Studi della Campania “Luigi Vanvitelli”, 80100 Naples, Italy; marco.carotenuto@unicampania.it

**Keywords:** vitamin E, neuroprotection mechanisms, neuroinflammation, central nervous system

## Abstract

Microglia are the first line of defense at the level of the central nervous system (CNS). Phenotypic change in microglia can be regulated by various factors, including the orexin system. Neuroinflammation is an inflammatory process mediated by cytokines, by the lack of interaction between neurotransmitters and their specific receptors, caused by systemic tissue damage or, more often, associated with direct damage to the CNS. Chronic activation of microglia could lead to long-term neurodegenerative diseases. This review aims to explore how tocopherol (vitamin E) and the orexin system may play a role in the prevention and treatment of microglia inflammation and, consequently, in neurodegenerative diseases thanks to its antioxidant properties. The results of animal and in vitro studies provide evidence to support the use of tocopherol for a reduction in microglia inflammation as well as a greater activation of the orexinergic system. Although there is much in vivo and in vitro evidence of vitamin E antioxidant and protective abilities, there are still conflicting results for its use as a treatment for neurodegenerative diseases that speculate that vitamin E, under certain conditions or genetic predispositions, can be pro-oxidant and harmful.

## 1. Introduction

Oxidative stress refers to a condition caused by the imbalance between oxidants and antioxidants in a biological system [1]. Reactive oxygen species (ROS) are secondary metabolites and arise from various essential biological processes, such as mitochondrial respiration, yet they are potentially harmful to cells. Consequently, eukaryotic organisms present various antioxidant defenses to avoid possible damage induced by ROS. They include, for example, superoxide dismutase (SOD) and catalase, glutathione peroxidase (Gpx) and peroxyroxins, as well as non-enzymatic factors including glutathione, flavonoids and vitamins [2,3]. Oxidative stress occurs when the enzymatic defenses fail to counteract the production of ROS causing damage. The result of this imbalance is an excess of ROS or a malfunction of the antioxidant system [4]. Although oxygen is an essential building block for life and is involved in signal transduction, gene transcription and other cellular activities, it also has a deleterious effect on biomolecules when found in the form of free radicals and ROS [1]. Another key molecule, involved in oxidative stress, is nitric oxide (NO); it regulates the relaxation and proliferation of vascular smooth muscle cells, leukocyte adhesion, angiogenesis, platelet aggregation, thrombosis, vascular tone and hemodynamics [5,6]. At physiological concentrations in the body, ROS are regulators of various physiological functions. In a chronic state of oxidative stress, reactive species can become harmful because they oxidize proteins and lipids and can damage DNA [7,8,9]. Reactive species may also mediate signaling, leading to microglia and primary astrocyte activation [10], resulting in a high secretion of pro-inflammatory cytokines and chemokines [11,12,13]. Numerous studies report that there is a significant connection between ROS and neurodegenerative diseases as well as aging [14,15,16,17], further suggesting a significant role of ROS and oxidative stress through a deleterious effect on biomolecules, in particular, on proteins. For example, in Alzheimer’s Disease (AD), there is the deposition of protein aggregates, extracellular amyloid plaques (Aβ), intracellular tau (τ) or neurofibrillary tangles [18,19]; in Parkinson’s Disease (PD), there is an impairment of dopaminergic neurons (DA) [20]; in amyotrophic lateral sclerosis (ALS) and Huntington Disease (HD), the mechanisms are still uncertain, but the substantia nigra is thought to have elevated levels of oxidized lipids, proteins and DNA [21,22,23,24,25].

## 2. The Orexin System

The orexin system (also known as the hypocretin system) consists of a population of neurons located at the hypothalamic level with the function of producing neuropeptides involved in the various regulatory processes, mainly of sleep and arousal [26,27]. The HCRT gene encodes the neuropeptide precursor peptide hypocretin, also known as prepro-orexin [28] from which two mature neuropeptides, OXA and OXB, originate by proteolytic processing [26,27], which bind to the G protein-coupled receptors OX ${}_{1}$ receptor (OX1R) and OX ${}_{2}$ receptor (OX2R), respectively [29]. Both OXA and OXB bind to HcrtR2 (OX2R), while OXA preferentially binds to HcrtR1 (OX1R) [27,30]. A possible imbalance of the hypocretin system can be associated with multiple diseases [31], narcolepsy [32,33] and emotional disorders, such as depression [28,34]. Furthermore, and of fundamental importance, they play an important role in neuroprotection by inhibiting oxidative stress and the inflammatory response through their type 1 and 2 receptors (OX1R and OX2R). In particular, some studies show that treatment with orexin A reduces the secretions of IL-1β, IL-6 and IL-8, as well as the production of reactive oxygen species (ROS) [35], which is why hypocretins may play a direct role in neurodegenerative diseases including PD and AD, although these mechanisms are less well known [36]. The functions of orexins prove to be different because of the multiple pathways involved, one of the main functions of orexin is neuroprotection [37]. The orexin system could lead to a decrease in oxidative stress, reducing the likelihood of neurodegenerative diseases. Currently, several in vitro studies have shown that OXA promotes both neuronal survival and neuronal protection from death caused by oxidative and hypoxic stress [38]. There are still few studies on the role of orexin on oxidative stress. Among those analyzed [39,40,41], we find a study by Butterick et al. [39] that shows how, in a hypothalamic cell line, the use of OXA leads to changes in cell survival following oxidative stress, particularly after H_2_O_2_; this study, in fact, has demonstrated that OXA is a neuroprotective molecule, partly because it reduces caspase-committed apoptosis and lipid peroxidation. There are also further studies regarding the orexin system and the reduction in oxidative stress, such as s study by Wang et al., which demonstrates how a neuroblastoma cell line reduces its oxidative stress induced by H_2_O_2_ following pretreatment with OXA [42]. H_2_O_2_ treatment is commonly used to induce oxidative stress [43]. These results indicate that orexin-A protects neuroblastoma cells from H_2_O_2_-induced neurotoxicity; moreover, the treatment with H_2_O_2_ reduces the antioxidant activity of SOD. It has been seen that this was attenuated by the pretreatment with OXA, in line with a study by Bihamta et al. conducted on cardiomyocyte cells [44]. This is believed to be thanks to the ability of OXA to activate the PI3K/MEK1/2/ERK1/2 signaling pathway and to attenuate the H_2_O_2_-induced increase in apoptosis and decrease in SH-SY5Y cell viability [42]. In another set of experiments, Hah Y.S et al. showed how the level of ROS in TNF-α-induced fibroblast-like synoviocytes was found to be reduced in the presence of orexin A. It is thought to be related to the NF-κB pathway [45]. Therefore, the limiting effect of orexin A on the inflammatory response is exerted via the classic NF-κB signaling pathway. Consequently, OXA may be useful for treating neurodegenerative diseases associated with oxidative damage. However, further in vivo studies are needed to evaluate the clinical significance of OXA prior to its clinical use.

## 3. Orexin in Microglia Activation

Microglial cells are monocyte–macrophage cells resident in the brain. In brain tissue, under physiological conditions, microglia cells are found in a quiescent state throughout the brain parenchyma, accounting for about 15% of the cell population [46], they are responsible for maintaining brain homeostasis by ensuring and controlling neuronal tropism [47]. At the morphological level, the microglia have narrow soma and long dynamic branches, which act as sentinels of the surrounding microenvironment. After appropriate stimulation, the microglial cells continue the differentiation process, previously interrupted to maintain the state of quiescence, to become immunocompetent phagocytic cells, that is, they undergo a rapid activation assuming an amoeboid form [48]. There may be different phenotypes of activation of microglia, characterized by a multiplicity of responses such as the phagocytosis, migration, proliferation and release of bioactive molecules [49]. In particular, it is possible to distinguish the classic M1-type phenotype (classic activation) associated with the production of pro-inflammatory cytokines, and the alternative M2-type phenotype (alternative activation), associated with the production of anti-inflammatory cytokines [50,51]. In conditions of acute inflammation, M1 cells are activated by LPS/IFN-γ and increase pro-inflammatory mediators, including IL-1β, IL-6, ROS, iNOS and TNF-α, as well as IL-8 [52], inducing a state of inflammatory tolerance. The M2 activation state is induced by parasitic products or associated signals (IL-4 and IL-13) with a long-term function for resolution and repair [53,54,55,56,57]. In this step, cellular signaling occurs through the IL-4Rα receptor that determines the inhibition of the NF-κB signaling produced by the activation of M1. In fact, M2 macrophages facilitate the resolution of inflammation through anti-inflammatory factors (for example IL-10, IL-13, TGF-β, VEGF, EGF, Arg1) to deactivate cellular phenotypes of inflammation and restoring homeostasis [54,58,59,60]. Increased levels of cytokines and chemokines, prostaglandin (PG) and prostaglandins E2 (PGE2), ROS and reactive nitrogen species, produced during inflammatory responses, lead to blood–brain barrier (BBB) damage, resulting in further cell damage but also a loss of neuronal function [61]. This type of activation is observed after brain injury or infection, as well as during the development of neuropathies such as AD, stroke or demyelinating diseases such as multiple sclerosis (MS), or even in the event that there is a lack of cells mediated by communication receptors. Consequently, in response to stress, injury or absence of receptor interaction [62], microgliocytes assume a pro-inflammatory phenotype that could lead to profound neuronal damage if uncontrolled or dysregulated [61,63]. Therefore, maintaining homeostasis at the microglial level is important. The orexin system also contributes to this, in fact, recent studies affirm that they act by modulating microglia, playing a fundamental role in neuroprotection [64,65]. Therefore, a reduction in the quantity of orexins could lead to an increase in microglial dysfunction with a consequent increase in the probability of developing neurodegenerative diseases [66]. As mentioned, the orexin systems play a pivotal role in neuroprotection and neuroinflammation [67,68]. OXA and OXB regulate the homeostatic mechanisms of energy balance and metabolism [64] through the activation of two G protein- coupled receptors (OX1R and OX2R, respectively) [68]. Recent studies in neuronal cell cultures have shown that orexin plays a role in neuroprotection [64,65] by reducing lipid peroxidation, apoptosis, and neuronal inflammation [39,69,70,71], in that the OX1R and OX2R receptors are both widely distributed on the cell membrane of brain tissue, but at the same time have also been found on the microglial cell membrane [72]. The data suggest that the neuroprotective effects of orexin, especially OXA, might be based on the modulation of microglia, the brain’s resident immune cells. In fact, recent studies have shown how OXA could play a fundamental role in neuroprotection, in part by reducing apoptosis and inflammation, thanks to its microglia modulation action [73,74]. As previously mentioned, microglia are the first line of defense of the brain environment capable of initiating adequate neuroinflammatory responses by transitioning from proinflammatory (M1) neurotoxic phenotypes to anti-inflammatory (M2) neuroprotective phenotypes. Although inflammatory processes may represent a physiological immune response required in certain contexts, chronic proinflammatory (M1) activation could be harmful, contributing to neuronal dysfunction and damage [75]. Numerous evidences, therefore, show that the involvement of the orexin/receptor system is fundamental in this process. In fact, in vivo for example, OXA would have shown neuroprotective actions in different contexts of focal cerebral ischemia in rodents, through the direct implication of mechanisms guided by microglia [38,39]. Furthermore, in another study by Xiong et al. [73], it has been shown that, under normal circumstances, the proinflammatory agent LPS determines both the increase in TNF- α production in BV-2 microglial cells and the expression of OX1R. Interestingly, pretreatment with OXA in the BV-2 cell line, prior to LPS stimulation, has been reported to lead to a reduction in TNF- α [73] IL-6 and inducible mediators of nitric oxide synthase (iNOS), thanks to greater expression of the M2 marker arginase-1 at the microglial level [74]. Further, many studies affirm and demonstrate that CaMKK β -activated AMPK (p-AMPK) is a fundamental process in the modulation and reduction of inflammation of the microglia [76,77]. A study by Wu et al. shows that AMPK could be activated by OXA [78], i.e., OXA would act on its receptors to activate CaMKK β , which in turn would activate AMPK. Activation of this factor would suppress further activation of inflammatory factors. In fact, as shown by the results derived from western blot studies, it is confirmed that treatment with OXA activated the p-AMPK pathway by reducing the expression of the factor p-NF κ B, and the cytokines IL-1 β and TNF- α , and by upregulating the production of anti-inflammatory cytokines such as IL-4 and Il-10 [66]. Therefore, despite the few studies in the literature that would require further study, it has been shown that orexin-deficient mice show a greater microglia response [73,79]. These data, support the idea that the neuropeptide OXA can act as an important immunoregulator of microglia, determining the reduction of proinflammatory cytokines and the increase of anti-inflammatory cytokines, thus promoting beneficial effects in the neuronal microenvironment [39].

## 4. Vitamin E and Microglia Mediated Neuroprotection

Vitamin E is a fat-soluble vitamin that plays the role of antioxidant against oxidative stress. As summarized in Table 1, the following eight isoforms of vitamin E have been identified: α-, β-, γ- and δ-tocopherol (αTOC, βTOC, γTOC and δTOC) and α-, β-, γ- and δ-tocotrienol (αT3, βT3, γT3 and δT3) [80,81]. The most studied isoform of vitamin E is α-Tocopherol (αTOC). The difference between the two major groups is the presence of an unsaturated side chain with three double bonds in the farnesyl isoprenoid tail of TOCs and an isoprenoid tail with three double bonds in T3s [82,83]; moreover, α-, β-, γ- and δ- are differentiated by the number and position of methyl groups on the chromanol ring. Vitamin E is known for its antioxidant properties and for its role in neuroplasticity, which could explain its neuroprotective effects. An “antioxidant” is any substance that can protect against oxidative stress damage caused by free radicals. Older cells decrease their ability to prevent and reduce oxidative damage. Thus, cellular senescence is associated with increased levels of oxidants, decreased body defenses against ROS and decreased effectiveness of repair mechanisms; factors that result in increased end products of oxidative damage [82]. The neuroprotective role of vitamin E in the brain, therefore, has been linked to neurogenesis, neuronal differentiation, hippocampal synaptic function and cell signaling pathways [83]. Antioxidants act in the following two ways: they prevent neuronal death due to oxidative stress (scavenging free radicals and, thus, preventing lipid peroxidation) and reduce the activation of transcription factors [84]. Transcription factors (themselves activated by oxidative stress) [85] are involved in the control of nerve cell survival and antioxidant-induced neuroprotection, although, to date, this mechanism is not fully understood [86]. Tocopherol is the most effective fat-soluble antioxidant, breaking chain reactions initiated by free radicals between Polyunsaturated fatty acids (PUFAs) in biological membranes, i.e., counteracting free radical reactivity by donating a hydrogen atom from an intact hydroxyl group to the free radical, thus stabilizing it [87]. Each tocopherol molecule can donate two electrons [88] before being “consumed”; the tocopherol molecule is then reduced to its previous state and can then be reused. Importantly, this reduction process is most likely carried out by ascorbic acid [88], which is why there are many studies reporting the antioxidant capacity of vitamin E linked to vitamin C [89]. A combination of different antioxidants, therefore, may offer additional benefit [90] because antioxidants may together have different protective effects. The long-term treatment with vitamin E tends to increase the concentration of tocopherol in the brain over time, thus, increasing its effectiveness as an antioxidant [91]. Clinical studies suggest that vitamin E would play an important role in the prevention of neurodegenerative diseases [92] thanks to its ability to act at the level of the microglia, causing a reduction in its activation, reducing inflammation [93,94,95]. This review aims to explore the pivotal role that tocopherol and the orexin system may play in the prevention and treatment of microglia inflammation and, consequently, in neuro-degenerative diseases.

The neuroprotective roles of vitamin E have been well documented in both in vivo and in vitro studies [91,94,95,96]. As antioxidants, tocopherols and tocotrienols protect tissue lipids from free radicals by reducing chemical species such as peroxyl, hydroxyl and superoxide radicals and singlet oxygen. Vitamin E has often been referred to as nature’s best chain antioxidant. Typically, one molecule of the vitamin protects about 100 membrane phospholipids [107]. In vivo, vitamin E and other endogenous antioxidants work in concert or synergistically by maintaining a reduced environment [108]. The effects of vitamin E on microglial cells have been studied in the short term, most studies, in line with the idea that microglial activation is a harmful process, have shown that vitamin E suppresses inflammatory activation of microglia, thus providing some neuroprotection [93,109]. Recent studies highlight how vitamin E is able to improve the various vital functions of N9 microglial cells; this includes the enhancement of protein turnover, the regulation of oxidative activity, the amount of proinflammatory agents and the absorption and degradation of extracellular protein material [110,111,112]. These effects could be explained not only by pure antioxidant effects [113], but also by the role of vitamin E as a hormone-like substrate, as proposed by Azzi et al. [114]. As regards the various isoforms at the level of the central nervous system, alpha tocopherol is the most biologically active form; in fact, a-tocopherol reduces the radicals of intracellular peroxide induced by stimulation with LPS at the level of the microglia [115]. As far as other isoforms are concerned, for example, tocotrienols may offer a greater bioavailability than tocopherols because their unsaturated hydrocarbon tails allow for better penetration into fatty tissue such as the brain [116]. A drastic decrease in ROS production by α-tocopherol has already been demonstrated in macrophages and is related to the inhibition of protein kinase C (PKC) [117]. The inhibition of PKC leads to the inhibition of NADPH-oxidase assembly [118] and, thus, reduces the production of superoxide. α-tocotrienol protects neurons from a glutamate-induced death better than γ-tocotrienol [119]. Interestingly, this effect is not related to the differential uptake of δ-tocotrienols in cells: γ-tocotrienols are absorbed more efficiently by neurons than α-tocotrienol [120]. To directly show the involvement of vitamin E in neuroprotection through the modulation of microglial responses, several papers have treated microglia cells with vitamin E alone, or with LPS alone, or pre-treated with vitamin E and then stimulated with LPS [93], the most commonly used pro-inflammatory stimulus for microglia, both in vitro and in vivo. Various studies show that vitamin E significantly suppressed LPS-induced microglia activation by decreasing the associated NO production and induction of IL-1α, TNF-α and iNOS expression. Indeed, Li et al. showed that incubating cells with 50 μM of vitamin E for 24 h significantly inhibited LPS-induced NO production (68%) and also reduced the expression of IL-1α (89%), TNF -α (32%) and iNOS (55%) [96]. More specifically, it was seen that the δ-tocotrienol taken up by BV2 microglia was 71% retained in BV2 cells even 24 h after its removal. The inhibitory effects of δ-tocotrienol on NO production by BV2 microglia could be partly attributable to δ-tocotrienol retention, as inhibition continued 48 h after stimulation with LPS [121]. Indeed, it showed that although various isoforms of tocotrienol at various concentrations were able to reduce the NO produced by BV2, δ -tocotrienol has the most potent effect, reducing NO levels by approximately 50%, even after 48 h [116]. Some studies have reported that vitamin E reduces the expression of iNOS, on human monocytic cells [122]. Indeed, vitamin E inhibits the phosphorylation of p38 MAPK and the activity of NFκB [93]. The vitamin E-induced inhibition of microglial responses after stimulation with LPS is, therefore, linked to the suppression of activation of p38 MAPK and NFκB, both of which regulate cytokine and iNOS expression [93]. In addition, vitamin E has also been reported to interact with the cyclooxygenase-2 (COX-2) signaling pathway, which is linked to pro-inflammatory signals [123] in BV2 cells. In this regard, several studies, including seminal observations from the Ames laboratory [124,125], suggest that c-tocopherol possesses significant anti-inflammatory activities that are distinct from its classical free radical defense action. Both c-tocopherol and δ-tocotrienol are more potent than a-tocopherol in inhibiting the catalytic function of Cyclooxygenase (COX) in BV2 cells [124]. COX, particularly inducible COX-2, are key inflammatory enzymes that mediate the conversion of arachidonic acid to prostaglandin E2 (PGE2) [126]. Finally, among the protective effects, a recent publication demonstrated that δ-tocotrienol is able to inhibit inflammation activation and subsequent IL-1β production in iJ774 macrophages [95]. The production of IL-1b, a key cytokine that mediates the inflammatory response, was found to be significantly reduced in vitamin E-treated microglia after 7 days in vitro, confirming the results of previous studies [93,109]. These findings indicate that antioxidants can be used to mitigate cytokine expression in the brain and protect against damage due to microglia activation. Regarding the ability of tocopherol to modulate the cell signaling pathways, in vitro and in vivo models have shown that vitamin E lowers the inflammatory responses that induce activation of microglia [93,127]. The chronic activation of microglia probably plays an important role in neurodegenerative disorders related to oxidative stress such as PD, AD [128,129] and ALS [93,130]. Vitamin E would, thus, suppress the harmful activation of microglia, thus offering possible neuroprotection [109]. Despite this, there are some studies that claim that antioxidants and their radicals often undergo other side reactions that can be classified as pro-oxidants [131,132]. One of the mechanisms hypothesized by Miller et al. [128] is that vitamin E supplementation has a pro-oxidant effect. There is a potential for vitamin E compounds to act as pro-oxidants, particularly the tocopheroxyl radical [133]. When the concentration of the tocopheroxyl radical is high enough, several undesirable side reactions can occur, which, in turn, can initiate a chain reaction that increases lipid peroxidation. In suspensions of the low-density lipoproteins isolated from blood, vitamin E can accelerate the peroxidation of polyunsaturated fatty acids [134]. The pro-oxidant radicals of vitamin E analogues caused intracellular LPO, GSH oxidation and cytotoxicity, which were prevented by an antioxidant [135]. A pro-oxidant effect of high-dose vitamin E may explain the increased mortality observed in adults taking high-dose vitamin E supplements for more than one year [128]. This suggests that the current recommendations for the maximum vitamin E dosage of 1000 mg/day of any form of α-tocopherol supplement (corresponding to synthetic vitamin E 1100 IU/day or natural vitamin E 1500 IU/day) should be revised in light of the new data [136].

## 5. Vitamin E in the Orexin System

There is still little evidence for this concept, but several studies correlate vitamin E with the hypocretin system. For example, a work conducted by Sanita Masoudi et al. studies the effect of an AS03 adjuvanted influenza vaccine (Pandemrix) against H1N1 swine flu. ASO3, a squalene-based immunological adjuvant used in various vaccine products, also contains α-tocopherol [133]. It has been hypothesized that α-tocopherol, through Nrf2 activation in neuronal cells, affects hypocretin expression and turnover. Factor 2, related to nuclear erythroid factor 2 (NRF2), is a transcription factor [137]. Nrf2 triggers the expression of cytoprotective genes, i.e., the active catalytic subunits of the constitutive proteasome, by binding to the antioxidant response element (ARE) [138]. Activating this pathway protects cells from oxidative stress-induced cell death. Increased oxidative stress leads to the death of neuronal cells during the pathogenesis of various chronic neurodegenerative diseases, such as AD, PD, HD and amyotrophic lateral sclerosis [139]. It was found that hypocretin also has an ARE site in its promoter region and could, therefore, be activated in a Nrf2-dependent manner [140]. It has been observed that the α-tocopherol AS03 component activates Nrf2 in neuronal cells that leads, on the one hand, to a greater expression of hypocretin with the consequent turnover of hypocretin and the formation of many hypocretin-specific peptides in the cells [140]. This could be achieved in two ways. On the other hand, α-tocopherol can freely cross the blood–brain barrier by increasing the amount of hypocretin with a consequent increase in the level of the educt. Moreover, α-tocopherol could influence the turnover of hypocretin resulting in high hypocretin fragments (the product). This underlines the potential of α-tocopherol to impart the increased formation of hypocretin fragments. This indicates that α-tocopherol has the potential to increase the amount of specific hypocretin fragments by increasing the expression of hypocretin and, in parallel, increasing the turnover of de novo synthesized hypocretin. However, in cases of a particular genetic predisposition, the use of vitamin E could be associated with an increase in narcolepsy [140]. Narcolepsy is closely associated with a specific human leukocyte antigen (HLA) allele. Of patients with narcolepsy, 90–100% carry HLA-DQ0602, a heterodimer encoded by DQA1 * 0102 (α chain) and DQB1 * 0602 (β chain) [137,138]. Due to this high association with HLA-DQ0602, narcolepsy is thought to be caused by an autoimmune-mediated process [139,140]. However, it is assumed that T cell mediated autoimmunity affects hypocretin neurons and could lead to the loss of hypocretin and, thus, to the development of narcolepsy [141,142]. In cases of genetic predisposition (DQB1 * 602), the hypocretin specific peptides/fragments are presented with greater affinity to the HLA subtype DQB1 * 602 on the cell surface and recognized by the immune system as foreign, which ultimately leads to the destruction of the respective cells. However, tocopherol by itself is not considered inductive to narcolepsy. It must be considered as an important factor in a multifactorial process, such as the interaction between genetic predisposition (DQB1 * 602), and the abrupt increase in the concentration of tocopherol [133].

**Figure 1:**
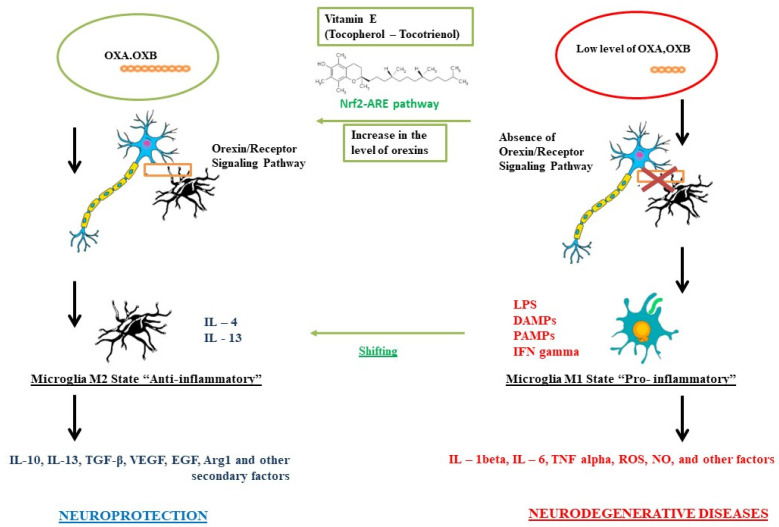
Role of vitamin E on the orexinergic system for neuroprotection.

As summarized in Figure 1, a possible imbalance of the hypocretin system with a consequent decrease in the interaction between orexins and its receptors, could reduce its neuroprotective action against the microglia, causing it to trigger a proinflammatory M1 phenotype, with the consequent formation of interleukins, cytokines, and ROS typical of an inflammatory response, which could lead to a greater probability of developing neurodegenerative diseases. However, a correct interaction between orexins and their receptors determines an anti-inflammatory response of the microglia towards an M2 state and the production of typical secondary metabolites, providing neuroprotection. We have seen how the use of vitamin E can act directly both at the level of microglia, causing a shift from the M1 state to the M2 state, but also at the level of the orexinergic system [138,139,140,141,142,143]. Despite the few studies published to date, it is hypothesized that vitamin E is involved in the activation of the NRF2/ARE pathway, which appears to be linked to the orexin system, causing an increase in turnover and formation, thus providing greater neuroprotection.

## 6. Conclusions and Future Scenarios

This mini review provides further support for the potential protective effects of vitamin E on neuroinflammation, the orexin system and its correlation with neurodegenerative diseases. Therefore, vitamin E could help reduce the chances of developing neurodegenerative diseases thanks to its fundamental role on microglia and its correlation with the orexin system. Further studies need to be conducted to better understand the mechanisms involved in the neuroprotective role of vitamin E and the benefit of its integration in our diet.

## Figures and Tables

**Table 1:** Biological activities of each type of vitamin E.

Type of Vitamin E	Biological Activity	Study Model	References
α-tocopherol	Reduces astrocytosis and microglia activation	Cell rat hippocampus	Ambrogini et al. [86]
α-tocopherol	Inhibits Microglia Activation	Pheochromocytoma cell line: PC12 cells	Li et al. [96]
α-tocopheryl acetate	Increases microglial activation and RAGE expression	Astroglial cell of mice	Bialowas-McGoey et al. 2008 [97]
α-tocopherol	Blocks glutamate release	Sprague Dawley rats	Barger et al. 2007 [98]
α-tocopherol	Attenuates expression of COX-2 and the production of proinflammatory cytokines	Cell rat hippocampus	Annàhazi et al. 2007 [99]
α-tocopherol	Reduces proinflammatory cytokines and production of ROS	Primary glial cultures	Stolzing et al. [100]
α-tocopherol	Decreases lipid peroxidation and IL-6 secretion	BALB/c mice	Godbout et al., 2004 [101]
Tocotrienols	Prevents death of HT4 cells treated with glutamate	HT4 hippocampal neuronal cells	Sen et al., 2000 [102]
δ-tocotrienol	Reduces NO production and IL-1β expression, inhibits PGE2 expression	BV2 microglia cells	Tan et al., 2020 [103]
α-, γ- and δ-tocotrienol	Reduce NO release	BV2 microglia cells	Tan et al., 2011 [104]
α-tocopherol	Attenuates COX-2 protein synthesis	BV2 microglia cells	Egger et al. [105]
γ-Tocopherol	Inhibits cyclooxygenase activity and nitrite accumulation	Murine RAW264.7 macrophages	Jiang Q et al., 2000 [106]

## Data Availability

Data sharing is not applicable to this article.

## References

[1] Sessa F., Messina G., Russo R., Salerno M., Castruccio Castracani C., Distefano A., Li Volti G., Calogero A.E., Cannarella R., Mongioi’ L.M. (2020). Consequences on aging process and human wellness of generation of nitrogen and oxygen species during strenuous exercise. Aging Male.

[2] Ott M., Gogvadze V., Orrenius S., Zhivotovsky B. (2007). Mitochondria, oxidative stress and cell death. Apoptosis.

[3] Calabrese V., Cornelius C., Mancuso C., Pennisi G., Calafato S., Bellia F., Bates T.E., Giuffrida Stella A.M., Schapira T., Dinkova Kostova A.T. (2008). Cellular stress response: A novel target for chemoprevention and nutritional neuroprotection in aging, neurodegenerative disorders and longevity. Neurochem. Res..

[4] Chiurchiù V., Orlacchio A., Maccarrone M. (2016). Is Modulation of Oxidative Stress an Answer? The State of the Art of Redox Therapeutic Actions in Neurodegenerative Diseases. Oxid Med. Cell Longev..

[5] Zheng M., Storz G. (2000). Redox sensing by prokaryotic transcription factors. BioChem. Pharmacol..

[6] Schiavone S., Neri M., Mhillaj E., Pomara C., Trabace L., Turillazzi E. (2016). The role of the NADPH oxidase derived brain oxidative stress in the cocaine-related death associated with excited delirium: A literature review. Toxicol. Lett..

[7] Neri M., Riezzo I., Pomara C., Schiavone S., Turillazzi E. (2016). Oxidative-Nitrosative Stress and Myocardial Dysfunctions in Sepsis: Evidence from the Literature and Postmortem Observations. Mediat. Inflamm..

[8] Zammit C., Muscat R., Sani G., Pomara C., Valentino M. (2015). Cerebral white matter injuries following a hypoxic/ischemic insult during the perinatal period: Pathophysiology, prognostic factors, and future strategy of treatment approach. A minireview. Curr. Pharm. Des..

[9] Turillazzi E., Neri M., Cerretani D., Cantatore S., Frati P., Moltoni L., Busardò F.P., Pomara C., Riezzo I., Fineschi V. (2016). Lipid peroxidation and apoptotic response in rat brain areas induced by long-term administration of nandrolone: The mutual crosstalk between ROS and NF-kB. J. Cell Mol. Med..

[10] Pawate S., Shen Q., Fan F., Bhat N.R. (2004). Redox regulation of glial inflammatory response to lipopolysaccharide and interferongamma. Neurosci. Res..

[11] Schiavone S., Mhillaj E., Neri M., Morgese M.G., Tucci P., Bove M., Valentino M., Di Giovanni G., Pomara C., Turillazzi E. (2017). Early Loss of Blood-Brain Barrier Integrity Precedes NOX2 Elevation in the Prefrontal Cortex of an Animal Model of Psychosis. Mol. Neurobiol..

[12] Neri M., Cantatore S., Pomara C., Riezzo I., Bello S., Turillazzi E., Fineschi V. (2011). Immunohistochemical expression of proinflammatory cytokines IL-1β, IL-6, TNF-α and involvement of COX-2, quantitatively confirmed by Western blot analysis, in Wernicke’s encephalopathy. Pathol. Res. Pract..

[13] Cerretani D., Bello S., Cantatore S., Fiaschi A.I., Montefrancesco G., Neri M., Pomara C., Riezzo I., Fiore C., Bonsignore A. (2011). Acute administration of 3,4-methylenedioxymethamphetamine (MDMA) induces oxidative stress, lipoperoxidation and TNFα-mediated apoptosis in rat liver. Pharm. Res..

[14] Von Arnim C.A., Gola U., Biesalski H.K. (2010). More than the sum of its parts? Nutrition in Alzheimer’s disease. Nutrition.

[15] Hung C.W., Chen Y.C., Hsieh W.L., Chiou S.H., Kao C.L. (2010). Ageing and neurodegenerative diseases. Ageing Res. Rev..

[16] Mandel S., Grünblatt E., Riederer P., Gerlach M., Levites Y., Youdim M.B. (2003). Neuroprotective strategies in Parkinson’s disease: An update on progress. CNS Drugs.

[17] Yu Y.C., Kuo C.L., Cheng W.L., Liu C.S., Hsieh M. (2009). Decreased antioxidant enzyme activity and increased mitochondrial DNA damage in cellular models of Machado-Joseph disease. J. Neurosci. Res..

[18] Selkoe D.J. (2001). Alzheimer’s disease results from the cerebral accumulation and cytotoxicity of amyloid beta-protein. J. Alzheimers Dis..

[19] Mattson M.P. (2004). Pathways towards and away from Alzheimer’s disease. Nature.

[20] Dias V., Junn E., Mouradian M.M. (2013). The role of oxidative stress in Parkinson’s disease. J. Parkinsons.

[21] Bosco D.A., Fowler D.M., Zhang Q., Nieva J., Powers E.T., Wentworth P., Lerner R.A., Kelly J.W. (2006). Elevated levels of oxidized cholesterol metabolites in Lewy body disease brains accelerate alpha-synuclein fibrilization. Nat. Chem. Biol..

[22] Nakabeppu Y., Tsuchimoto D., Yamaguchi H., Sakumi K. (2007). Oxidative damage in nucleic acids and Parkinson’s disease. J. Neurosci. Res..

[23] Zeevalk G.D., Razmpour R., Bernard L.P. (2008). Glutathione and Parkinson’s disease: Is this the elephant in the room?. Biomed. Pharmacother..

[24] Puspita L., Chung S.Y., Shim J.W. (2017). Oxidative stress and cellular pathologies in Parkinson’s disease. Mol. Brain..

[25] Singh A., Kukreti R., Saso S., Kukreti S. (2019). Oxidative Stress: A Key Modulator in Neurodegenerative Diseases. Molecules.

[26] Polito R., Monda V., Nigro E., Messina A., Di Maio G., Giuliano M.T., Orrù S., Imperlini E., Calcagno G., Mosca L. (2020). The Important Role of Adiponectin and Orexin-A, Two Key Proteins Improving Healthy Status: Focus on Physical Activity. Front. Physiol..

[27] Polito R., Nigro E., Messina A., Monaco M.L., Monda V., Scudiero O., Cibelli G., Valenzano A., Picciocchi E., Zammit C. (2018). Adiponectin and orexin-A as a potential immunity link between Adipose tissue and central nervous system. Front. Physiol..

[28] Dong X.S., Ma S.F., Cao C.W., Li J., An P., Zhao L., Liu N.Y., Yan H., Hu Q.T., Mignot E. (2013). Hypocretin (orexin) neuropeptide precursor gene, HCRT, polymorphisms in early-onset narcolepsy with cataplexy. Sleep Med..

[29] Sperandeo R., Maldonato M.N., Messina A., Cozzolino P., Monda M., Cerroni F., Romano P., Salerno M., Maltese A., Roccella M. (2018). Orexin system: Network multi-tasking Acta. Med. Mediterr..

[30] Chieffi S., Carotenuto M., Monda V., Valenzano A., Villano I., Precenzano F., Tafuri D., Salerno M., Filippi N., Nuccio F. (2017). Orexin system: The key for a healthy life. Front. Neurol..

[31] El-Bachá R.S., De-Lima-Filho J.L., Guedes R.C. (1998). Dietary Antioxidant Deficiency Facilitates Cortical Spreading Depression Induced by Photoactivated Riboflavin. Nutr. Neurosci..

[32] Oliveira L.M., Henrique E., Bustelli I.B., Netto N.F.C., Moreira T.S., Takakura A.C., Caetano A.L. (2020). Depletion of hypothalamic hypocretin/orexin neurons correlates with impaired memory in a Parkinson’s disease animal model. Exp. Neurol..

[33] Mayo M.C., Deng J.C., Albores J., Zeidler M., Harper R.M., Avidan A.Y. (2015). Hypocretin Deficiency Associated with Narcolepsy Type 1 and Central Hypoventilation Syndrome in Neurosarcoidosis of the Hypothalamus. J. Clin. Sleep Med. JCSM Off. Publ. Am. Acad. Sleep Med..

[34] Mehr J.B., Mitchison D., Bowrey H.E., James M.H. (2021). Sleep dysregulation in binge eating disorder and “food addiction”: The orexin (hypocretin) system as a potential neurobiological link. Neuropsychopharmacology.

[35] Li H., Lu J., Li S., Huang B., Shi G., Mou T., Xu Y. (2021). Increased Hypocretin (Orexin) Plasma Level in Depression, Bipolar Disorder Patients. Front. Psychiatry.

[36] Sun M., Wang W., Li Q., Yuan T., Weng W. (2018). Orexin A may suppress inflammatory response in fibroblast-like synoviocytes. Minghui Biomed. Pharmacother..

[37] Zhan S., Che P., Zhao X., Li N., Ding Y., Liu J., Li S., Ding K., Han L., Huang Z. (2019). The molecular mechanism of tumor necrosis factor alpha regulates the expression, sleep and behavior of hypocretin (orexin). J. Cell Mol. Med..

[38] Harada S., Fujita-Hamabe W., Tokuyama S. (2011). Effect of orexin-A on post-ischemic glucose intolerance and neuronal damage. J. Pharmacol. Sci..

[39] Couvineau A., Voisin T., Nicole P., Gratio V., Abad C., Tan Y.V. (2019). Orexins as new therapeutic targets in inflammatory and neurodegenerative diseases. Front. Endocrinol..

[40] Butterick T.A., Nixon J.P., Billington C.J., Kotz C.M. (2012). Orexin A decreases lipid peroxidation and apoptosis in a novel hypothalamic cell model. Neurosci. Lett..

[41] Duffy C.M., Nixon J.P., Butterick T.A. (2016). Orexin A attenuates palmitic acid-induced hypothalamic cell death. Mol. Cell Neurosci..

[42] Wang L., He T., Wan B., Wang X., Zhang L. (2019). Orexin A ameliorates HBV X protein-induced cytotoxicity and inflammatory response in human hepatocytes. Artif. Cells Nanomed. Biotechnol..

[43] Wang C.M., Yang C.Q., Cheng B.H., Chen J., Bai B. (2018). Orexin-A protects SH-SY5Y cells from H_2_O_2_-induced oxidative damage via the PI3K/MEK1/2/ERK½. Int. J. Immunopathol. Pharm..

[44] Iloki-Assanga S.B., Lewis-Luján L.M., Fernández-Angulo D., Gil-Salido A.A., Lara-Espinoza C.L., Rubio-Pino J.L. (2015). Bucida buceras retino-protective effect against H_2_O_2_-induced oxidative stress in the human retinal pigment epithelial cell line. BMC Complement. Altern. Med..

[45] Bihamta M., Hosseini A., Ghorbani A., Boroushaki M.T. (2017). Protective effect of pomegranate seed oil against oxidative stress induced by H_2_O_2_ in cardiomyocytes. Avicenna J. Phytomed..

[46] Hah Y.S., Lee Y.R., Jun J.S., Lim H.S., Kim H.O., Jeong Y.G., Hur G.M., Lee S.Y., Chung M.J., Park J.W. (2010). A20 suppresses inflammatory responses and bone destruction in human fibroblast-like synoviocytes and in mice with collagen-induced arthritis. Arthritis Rheum..

[47] Dickson D.W., Lee S.C., Mattiace L.A., Yen S.H., Brosnan C. (1993). Microglia and cytokines in neurological disease, with special reference to AIDS and Alzheimer’s disease. Glia.

[48] Kettenmann H., Hanisch U.K., Noda M., Verkhratsky A. (2011). Physiology of microglia. Physiol. Rev..

[49] Kettenmann H., Kirchhoff F., Verkhratsky A. (2013). Microglia: New roles for the synaptic stripper. Neuron.

[50] Soulet D., Rivest S. (2008). Microglia. Curr. Biol..

[51] Lee J.Y., Jhun B.S., Oh Y.T., Lee J.H., Choe W., Baik H.H., Ha J., Yoon K.S., Kim S.S., Kang I. (2006). Activation of adenosine A3 receptor suppresses lipopolysaccharide-induced TNF-a production through inhibition of PI 3- kinase/Akt and NF-jB activation in murine BV2 microglial cells. Neurosci. Lett..

[52] Cherry J.D., Olschowka J.A., O’Banion M.K. (2014). Neuroinflammation and M2 microglia: The good, the bad, and the inflamed. J. Neuroinflammation.

[53] Nagy E.E., Frigy A., Szász J.A., Horváth E. (2020). Neuroinflammation and microglia/macrophage phenotype modulate the molecular background of post-stroke depression: A literature review. Exp. Med..

[54] Rutschman R., Lang R., Hesse M., Ihle J.N., Wynn T.A., Murray P.J. (2001). Cutting edge: STAT6-dependent substrate depletion regulates nitric oxide production. J. Immunol..

[55] Gordon S. (2003). Alternative activation of macrophages. Nat. Rev. Immunol..

[56] Lawrence T., Natoli G. (2011). Transcriptional regulation of macrophage polarization: Enabling diversity with identity. Nat. Rev. Immunol..

[57] Mills C.D. (2012). M1and M2 macrophages: Oracles of health and disease. Crit. Rev. Immunol..

[58] Wynn T.A., Chawla A., Pollard J.W. (2013). Macrophage biology in development, homeostasis and disease. Nature.

[59] Gordon S., Martinez F.O. (2010). Alternative activation of macrophages: Mechanism and functions. Immunity.

[60] Ortega-Gómez A., Perretti M., Soehnlein O. (2013). Resolution of inflammation: An integrated view. EMBO Mol. Med..

[61] Manich G., Recasens M., Valente T., Almolda B., González B., Castellano B. (2019). Role of the CD200-CD200R Axis During Homeostasis and Neuroinflammation. Neuroscience.

[62] Wang W.Y., Tan M.S., Yu J., Tan L. (2015). Role of proinflammatory cytokines released from microglia in Alzheimer’s disease. Ann. Transl. Med..

[63] Chen W.W., Zhang X., Huang W.J. (2016). Role of neuroinflammation in neurodegenerative diseases. Mol. Med. Rep..

[64] Aloisi F. (2001). Immune function of microglia. Glia.

[65] Block M.L., Zecca L., Hong J.S. (2007). Microglia-mediated neurotoxicity: Uncovering the molecular mechanisms. Nat. Rev. Neurosci..

[66] Li T., Xu W., Ouyang J., Lu X., Sherchan P., Lenahan C., Irio G., Zhang J.H., Zhao J., Zhang Y. (2020). Orexin A alleviates neuroinflammation via OXR2/CaMKKβ/AMPK signaling pathway after ICH in mice. J. Neuroinflammation.

[67] Sun H., He X., Tao X., Hou T., Chen M., He M., Liao H. (2020). The CD200/CD200R signaling pathway contributes to spontaneous functional recovery by enhancing synaptic plasticity after stroke. J. Neuroinflammation.

[68] Wang L., Yu C.C., Liu X.Y., Deng X.N., Tian Q., Du Y.J. (2021). Epigenetic Modulation of Microglia Function and Phenotypes in Neurodegenerative Diseases. Neural Plast..

[69] Yuan L.B., Dong H.L., Zhang H.P., Zhao R.N., Gong G., Chen X.M., Zhang L.N., Xiong L. (2011). Neuroprotective Effect of Orexin-A Is Mediated by an Increase of Hypoxia-inducible Factor-1 Activity in Rat. Anesthesiology.

[70] Becquet L., Abad C., Leclercq M., Miel C., Jean L., Riou G., Couvineau A., Boyer O., Tan Y.V. (2019). Systemic administration of orexin A ameliorates established experimental autoimmune encephalomyelitis by diminishing neuroinflammation. J. Neuroinflammation.

[71] Sokolowska P., Urbańska A., Biegańska K., Wagner W., Ciszewski W., Namiecińska M., Zawilska J.B. (2014). Orexins protect neuronal cell cultures against hypoxic stress: An involvement of Akt signaling. J. Mol. Neurosci..

[72] Synchikova A.P., Horiuchi H., Nabekura J. (2019). The effect of orexin A application on the reaction of microglia cells body size stimulated by LPS injection. Med. Acad. J..

[73] Xiong X., White R.E., Xu L., Yang L., Sun X., Zou B., Pascual C., Sakurai T., Giffard R.C., Xie X.S. (2013). Mitigation of murine focal cerebral ischemia by the hypocretin/orexin system is associated with reduced inflammation. Stroke.

[74] Duffy C.M., Yuan C., Wisdorf L.E., Billington C.J., Kotz C.M., Nixon J.P., Butterick T.A. (2015). Role of orexin A signaling in dietary palmitic acid-activated microglial cells. Neurosci. Lett..

[75] Perry V.H., Holmes C. (2014). Microglial priming in neurodegenerative disease. Nat. Rev. Neurol..

[76] Xu Y., Xu Y., Wang Y., Wang Y., He L., Jiang Z., Huang Z., Liao H., Li J., Saavedra J.M. (2015). Telmisartan prevention of LPS-induced microglia activation involves M2 microglia polarization via CaMKKβ-dependent AMPK activation. Brain Behav. Immun..

[77] Zhou X., Cao Y., Ao G., Hu L., Liu H., Wu J., Wang X., Jin M., Zheng S., Zhen X. (2014). CaMKKβ-dependent activation of AMP-activated protein kinase is critical to suppressive effects of hydrogen sulfide on neuroinflammation. Antioxid Redox Signal..

[78] Wu W.N., Wu P.F., Zhou J., Guan X.L., Zhang Z., Yang Y.J., Long L.H., Xie N., Chen J.G., Wang F. (2013). Orexin-A activates hypothalamic AMP-activated protein kinase signaling through a Ca^2+^-dependent mechanism involving voltage-gated L-type calcium channel. Mol. Pharmacol..

[79] Wang L., Liu Y., Yan S., Du T., Fu X., Gong X., Zhou X., Zhang T., Wang X. (2020). Disease Progression-Dependent Expression of CD200R1 and CX3CR1 in Mouse Models of Parkinson’s Disease. Aging Dis..

[80] Miyazawa T., Burdeos G.C., Itaya M., Nakagawa K., Miyazawa T. (2019). Vitamin E: Regulatory Redox Interactions. IUBMB Life.

[81] Mustacich D.J., Bruno R.S., Traber M.G. (2007). Vitamin E. Vitam. Horm..

[82] Wu J.H., Croft K.D. (2007). Vitamin E metabolism. Mol. Aspects Med..

[83] Kamal-Eldin A., Appelqvist L.A. (1996). The chemistry and antioxidant properties of tocopherols and tocotrienols. Lipids.

[84] Sen C.K., Khanna S., Roy S. (2006). Tocotrienols: Vitamin E beyond tocopherols. Life Sci..

[85] Floyd R.A., West M., Hensley K. (2001). Oxidative biochemical markers: Clues to understanding aging in long-lived species. Exp. Gerontol..

[86] Ambrogini P., Betti M., Galati C., Di Palma M., Lattanzi D., Savelli D., Galli F., Cuppini R., Minelli A. (2016). Alpha-Tocopherol and Hippocampal Neural Plasticity in Physiological and Pathological Conditions. Int. J. Mol. Sci..

[87] Lohr J.B., Kuczenski R., Niculescu A. (2003). Oxidative mechanisms and tardive dyskinesia. CNS Drugs.

[88] Altavilla D., Deodato B., Campo G.M., Arlotta M., Miano M., Squadrito G., Saitta A., Cucinotta D., Ceccarelli S., Ferlito M. (2000). A novel dual vitamin E-like antioxidant, inhibits activation of nuclear factor-kappaB and reduces the inflammatory response in myo cardial ischemia-reperfusion injury. Cardiovasc. Res..

[89] Behl C. (2000). Vitamin E protects neurons against oxidative cell death in vitro more effectively than 17-beta estradiol and induces the activity of the transcription factor NF-kappaB. J. Neural. Transm..

[90] Vatassery G.T. (1998). Vitamin E and other endogenous antioxidants in the central nervous system. Geriatrics.

[91] Wolf R., Wolf D., Ruocco V. (1998). Vitamin E: The radical protector. J. Eur. Acad. Derm. Venereol..

[92] Martin A., Youdim K., Szprengiel A. (2002). Roles of Vitamins E and C on Neurodegenerative Diseases and Cognitive Performance. Nutr. Rev..

[93] Delanty N., Dichter M.A. (2000). Antioxidant therapy in neurologic disease. Arch. Neurol..

[94] Grundman M. (2000). Vitamin E and Alzheimer disease: The basis for additional clinical trials. Am. J. Clin. Nutr..

[95] Takahashi T., Nakaso K., Horikoshi Y., Hanaki T., Yamakawa M., Nakasone M., Kitagawa Y., Koike T., Matsura T. (2016). Rice Bran Dietary Supplementation Improves Neurological Symptoms and Loss of Purkinje Cells in Vitamin E-Deficient Mice. Yonago Acta Med..

[96] Li Y., Liu L., Barger S.W. (2001). Vitamin E Suppression of Microglial Activation Is Neuroprotective. J. Neurosci. Res..

[97] Bialowas-McGoey L.A., Lesicka A., Whitaker-Azmitia P.M. (2008). Vitamin E increases S100B-mediated microglial activation in an S100B-overexpressing mouse model of pathological aging. Glia.

[98] Barger S.W., Goodwin M.E., Porter M.M., Beggs M.L. (2007). Glutamate release from activated microglia requires the oxidative burst and lipid peroxidation. J. Neurochem..

[99] Annaházi A., Mracskó E., Süle Z., Karg E., Penke B., Bari F., Farkas E. (2007). Pre-treatment and post-treatment with alpha-tocopherol attenuates hippocampal neuronal damage in experimental cerebral hypoperfusion. Eur. J. Pharmacol..

[100] Stolzing A., Widmer R., Jung T. (2006). Tocopherol-mediated modulation of age-related changes in microglial cells: Turnover of extracellular oxidized protein material. Free Radic. Biol. Med..

[101] Godbout J.P., Berg B.M., Kelley K.W. (2004). A-Tocopherol reduces lipopolysaccharide-induced peroxide radicalformation and interleukin-6 secretion in primary murine microglia and in brain. J. Neuroimmunol..

[102] Sen C.K., Khanna S., Roy S., Packer L. (2000). Molecular basis of vitamin E action. Tocotrienol potently inhibits glutamate-induced pp60(c-Src) kinase activation and death of HT4 neuronal cells. J. Biol. Chem..

[103] Tan S.W., Ali D.A., Khaza’ai H., Wong J.W., Vidyadaran S. (2020). Cellular uptake and anti-inflammatory effects of palm oil-derived delta (δ)- tocotrienol in microglia. Cell. Immunol..

[104] Tan S.W., Ramasamy R., Abdullah M. (2011). Inhibitory effects of palm a-, c- and d-tocotrienol on lipopolisaccaride- induced nitric oxide production in BV2 microglia. Cell Immunol..

[105] Egger T., Schuligoi R., Wintersperger A., Amann R., Malle E., Sattler W. (2003). Vitamin E (alpha-tocopherol) attenuates cyclo-oxygenase 2 transcription and synthesis in immortalized murine BV-2 microglia. Biochem. J..

[106] Jiang Q., Elson-Schwab I., Courtemance C., Ames B.N. (2000). Gammatocopherol and its major metabolite, in contrast to alpha-tocopherol, inhibit cyclooxygenase activity in macrophages and epithelial cells. Proc. Natl. Acad. Sci. USA.

[107] Behl C. (1999). Vitamin E and other antioxidants in neuroprotection. Int. J. Vitam. Nutr. Res..

[108] Goodman Y., Mattson M.P. (1994). Secreted forms of β-amyloid precursor protein protect hippocampal neurons against amyloid β-peptide-induced oxidative injury. Exp. Neurol..

[109] Halks-Miller M., Henderson M., Eng L.F. (1986). α-Tocopherol decreases lipid peroxidation, neuronal necrosis, and reactive gliosis in reaggregate cultures of fetal rat brain. J. Neuropathol. Exp. Neurol..

[110] The HOPE and HOPE-TOO Trial Investigators (2005). Effects of long-term vitamin E supplementation on cardiovascular events and cancer: A randomised controlled trial. JAMA.

[111] Yadav A., Kumari R., Yadav A., Mishra J.P., Srivatva S., Prabha S. (2016). Antioxidants and its functions in human body—A Review. Res. Environ. Life Sci..

[112] Heppner F.L., Roth K., Nitsch R., Hailer N.P. (1998). Vitamin E induces ramification and downregulation of adhesion molecole in cultures microglial cells. Glia.

[113] Grundmann M., Grundman M., Delaney P. (2002). Antioxidant strategies for Alzheimer’s disease. Proc. Nutr. Soc..

[114] Azzi A., Ricciarelli R., Zingg J.M. (2002). Non-antioxidant molecular functions of α-tocopherol (vitamin E). FEBS Lett..

[115] Tasinato A., Boscoboinik D., Bartoli G.M., Maroni P., Azzi A. (1995). d-Alpha-tocopherol inhibition of vascular smooth muscle cell proliferation occurs at physiological concentrations, correlates with protein kinase C inhibition, and is independent of its antioxidant properties. Proc. Natl. Acad. Sci. USA.

[116] Cachia O., Benna J.E., Pedruzzi E., Descomps B., Gougerot-Pocidalo M.A., Leger C.L. (1998). Alpha-tocopherol inhibits the respiratory burst in human monocytes. Attenuation of p47(phox) membrane translocation and phosphorylation. J. Biol. Chem..

[117] Kamat J.P., Devasagayam T.P. (1995). Tocotrienols from palm oil as potent inhibitors of lipid peroxidation and protein oxidation in rat brain mitochondria. Neurosci. Lett..

[118] Lively S., Schlichter L.C. (2018). Microglia Responses to Pro-inflammatory Stimuli (LPS, IFNγ+TNFα) and Reprogramming by Resolving Cytokines (IL-4, IL-10). Front. Cell Neurosci..

[119] Lee G.Y., Han S.N. (2018). The Role of Vitamin E in Immunity. Nutrients.

[120] Jiang Q., Lykkesfeldt J., Shigenaga M.K., Shigeno E.T., Christen S., Ames B.N. (2002). Gamma-tocopherol supplementation inhibits protein nitration and ascorbate oxidation in rats with inflammation. Free Rad. Biol. Med..

[121] Funk C.D. (2001). Prostaglandins and leukotrienes: Advances in eicosanoid biology. Science.

[122] Wang S.W., Yang S.G., Liu W., Zhang Y.X., Xu P.X., Wang T., Ling T.J., Liu R.T. (2016). Alpha-tocopherol quinine ameliorates spatial memory deficits by reducing beta-amyloid oligomers, neuroinflammation and oxidative stress in transgenic mice with Alzheimer’s disease. Behav. Brain Res..

[123] De Rijk M.C., Breteler M.M., den Breeijen J.H., Launer L.J., Grobbee D.E., van der Meche F.G. (1997). Dietary antioxidants and Parkinson disease. The Rotterdam study. Arch. Neurol..

[124] Sano M., Ernesto C., Thomas R.G., Klauber M.R., Schafer K., Grundman M., Woodbury P., Growdon J., Cotman C.W., Pfeiffer E. (1997). A controlled trial of selegiline, α-tocopherol, or both as treatment for Alzheimer’s disease. The Alzheimer’s disease cooperative study. N. Engl. J. Med..

[125] Ascherio A., Weisskopf M.G., O’Reilly E.J., Jacobs E.J., McCullough M.L., Calle E.E., Cudkowicz M., Thun M.J. (2005). Vitamin E intake and risk of amyotrophic lateral sclerosis. Ann. Neurol..

[126] Bowry V., Stocker R. (1993). Tocopherol-mediated peroxidation: The prooxidant effect of vitamin E on the radical-initiated effect oxidation of human low-density lipoprotein. J. Am. Chem. Soc..

[127] Pearson P.J., Lewis S.A., Britton J., Fogarty A. (2004). Vitamin E supplementation in the treatment of asthma: A randomised controlled trial. Thorax.

[128] Miller E.R., Pastor-Barriuso R., Dalal D., Riemersma R.A., Appel L.J., Guallar E. (2005). Meta-analysis: High dose vitamin E supplementation may increase all-cause mortality. Ann. Intern. Med..

[129] DeLong J., Prange R.K., Hodges D.M., Forney C.F., Bishop M.C., Quilliam M. (2002). Using a modified ferrous-oxidationxyelnol orange (FOX) assay for detection of lipid hydrperoxides in plant tissue. J. Agric. Food Chem..

[130] Balluz L., Kieszak S., Philen R.M., Mulinare J. (2000). Vitamin and mineral supplement use in the United States. Arch. Fam. Med..

[131] Tafazoli S., Wright J.S., O’Brien P.J. (2005). Prooxidant and Antioxidant Activity of Vitamin E Analogues and Troglitazone. Chem. Res. Toxicol..

[132] Institute of Science (2000). Dietary Reference Intakes for Vitamin C, Vitamin E, Selenium and Carotenoids.

[133] Masoudi S., Ploen D., Kunz K., Hildt E. (2014). The adjuvant component α-tocopherol triggers the expression and turnover of hypocretin in vitro and its implications in the development of narcolepsy through the modulation of Nrf2. Vaccine.

[134] Moi P., Chan K., Asunis I., Cao A., Kan Y.W. (1994). Isolation of NF-E2-related factor 2 (Nrf2), a NF-E2-like basic leucine zipper transcriptional activator that binds to the tandem NF-E2/AP1 repeat of the beta-globin locus control region. Proc. Natl. Acad. Sci. USA.

[135] Johnson J.A., Johnson D.A., Kraft A.D., Calkins M.J., Jakel R.J., Vargas M.R., Chen P. (2008). The Nrf2-ARE pathway: An indicator and modulator of oxidative stress in neurodegeneration. Ann. N. Y. Acad. Sci..

[136] Masoudi S., Ploen D., Hildt E. (2015). Is there an association between pandemic influenza H1N1 vaccination and the manifestation of narcolepsy?. J. Vaccines Vaccin..

[137] Matsuki K., Grumet F.C., Lin X., Gelb M., Guilleminault C., Dement W.C., Mignot E. (1992). DQ (rather than DR) gene marks susceptibility to narcolepsy. Lancet.

[138] Nishino S., Mignot E. (1997). Pharmacological aspects of human and canine narcolepsy. Prog. Neurobiol..

[139] Nishino S., Okuro M., Kotorii N., Anegawa E., Ishimaru Y., Matsumura M., Kanbayashi T. (2010). Hypocretin/orexin and narcolepsy: New basic and clinical insights. Acta Physiol..

[140] Mahlios J., De la Herrán-Arita A.K., Mignot E. (2013). The autoimmune basis of narcolepsy. Curr. Opin. Neurobiol..

[141] De la Herrán-Arita A.K., García-García F. (2014). Narcolepsy as an immune-mediated disease. Sleep Disord..

[142] LAKEMEDELSVERKET. http://www.lakemedelsverket.se/upload/nyheter/2011/Fallinventeringsrport_pandermrix_110630.pdf.

[143] Sessa F., Maglietta F., Bertozzi G., Salerno M., Di Mizio G., Messina G., Montana A., Ricci P., Pomara C. (2019). Human brain injury and mirnas: An experimental study. Int. J. Mol. Sci..

