# Correction: La Torre et al. Role of Vitamin E and the Orexin System in Neuroprotection. *Brain Sci.* 2021, *11*, 1098

**DOI:** 10.3390/brainsci12121709

**Published:** 2022-12-13

**Authors:** Maria Ester La Torre, Ines Villano, Marcellino Monda, Antonietta Messina, Giuseppe Cibelli, Anna Valenzano, Daniela Pisanelli, Maria Antonietta Panaro, Nicola Tartaglia, Antonio Ambrosi, Marco Carotenuto, Vincenzo Monda, Giovanni Messina, Chiara Porro

**Affiliations:** 1 Department of Clinical and Experimental Medicine, University of Foggia, 71122 Foggia, Italy; 2 Department of Experimental Medicine, Section of Human Physiology and Unit of Dietetics and Sports Medicine, Università degli Studi della Campania “Luigi Vanvitelli”, 80100 Naples, Italy; 3 Department of Biosciences, Biotechnologies and Biopharmaceutics, University of Bari, 70125 Bari, Italy; 4 Department of Medical and Surgical Sciences, University of Foggia, Viale Pinto, 71122 Foggia, Italy; 5 Clinic of Child and Adolescent Neuropsychiatry, Department of Mental Health, Physical and Preventive Medicine, Università degli Studi della Campania “Luigi Vanvitelli”, 80100 Naples, Italy

## 1. General Changes

In the original article [1], there was a mistake about CD200-CD200R interaction and orexin nomenclature. The changes listed below are changes and new additions to the original article content.

## 2. Specific Changes

Abstract: “by the lack of interaction between neurotransmitters and their specific receptors.”

Section 2. The Orexin System: “The orexin system (also known as the hypocretin system) consists of a population of neurons located at the hypothalamic level with the function of producing neuropeptides involved in the various regulatory processes, mainly of sleep and arousal [26,27]. The HCRT gene encodes the neuropeptide precursor peptide hypocretin, also known as prepro-orexin [28] from which two mature neuropeptides, OXA and OXB, originate by proteolytic processing [26,27], which bind to the G protein-coupled receptors OX_1_ receptor (OX1R) and OX_2_ receptor (OX2R), respectively [29]”.

Section 3. Orexin in Microglia Activation: “through the activation of two G protein- coupled receptors (OX1R and OX2R, respectively) [68]. Recent studies in neuronal cell cultures have shown that orexin plays a role in neuroprotection [64,65] by reducing lipid peroxidation, apoptosis, and neuronal inflammation [39,69–71], in that the OX1R and OX2R receptors are both widely distributed on the cell membrane of brain tissue, but at the same time have also been found on the microglial cell membrane [72]. The data suggest that the neuroprotective effects of orexin, especially OXA, might be based on the modulation of microglia, the brain’s resident immune cells. In fact, recent studies have shown how OXA could play a fundamental role in neuroprotection, in part by reducing apoptosis and inflammation, thanks to its microglia modulation action [73,74]. As previously mentioned, microglia are the first line of defense of the brain environment capable of initiating adequate neuroinflammatory responses by transitioning from proinflammatory (M1) neurotoxic phenotypes to anti-inflammatory (M2) neuroprotective phenotypes. Although inflammatory processes may represent a physiological immune response required in certain contexts, chronic proinflammatory (M1) activation could be harmful, contributing to neuronal dysfunction and damage [75]. Numerous evidences, therefore, show that the involvement of the orexin/receptor system is fundamental in this process. In fact, in vivo for example, OXA would have shown neuroprotective actions in different contexts of focal cerebral ischemia in rodents, through the direct implication of mechanisms guided by microglia [38,39]. Furthermore, in another study by Xiong et al. [73], it has been shown that, under normal circumstances, the proinflammatory agent LPS determines both the increase in TNF-α production in BV-2 microglial cells and the expression of OX1R. Interestingly, pretreatment with OXA in the BV-2 cell line, prior to LPS stimulation, has been reported to lead to a reduction in TNF-α [73] IL-6 and inducible mediators of nitric oxide synthase (iNOS), thanks to greater expression of the M2 marker arginase-1 at the microglial level [74]. Further, many studies affirm and demonstrate that CaMKKβ-activated AMPK (p-AMPK) is a fundamental process in the modulation and reduction of inflammation of the microglia [76,77]. A study by Wu et al. shows that AMPK could be activated by OXA [78], i.e., OXA would act on its receptors to activate CaMKKβ, which in turn would activate AMPK. Activation of this factor would suppress further activation of inflammatory factors. In fact, as shown by the results derived from western blot studies, it is confirmed that treatment with OXA activated the p-AMPK pathway by reducing the expression of the factor p-NFκB, and the cytokines IL-1β and TNF-α, and by upregulating the production of anti-inflammatory cytokines such as IL-4 and Il-10 [66]. Therefore, despite the few studies in the literature that would require further study, it has been shown that orexin-deficient mice show a greater microglia response [73,79]. These data, support the idea that the neuropeptide OXA can act as an important immunoregulator of microglia, determining the reduction of proinflammatory cytokines and the increase of anti-inflammatory cytokines, thus promoting beneficial effects in the neuronal microenvironment [39]”.

Section 5. Vitamin E in the Orexin System, Paragraph 2: “As summarized in Figure 1, a possible imbalance of the hypocretin system with a consequent decrease in the interaction between orexins and its receptors, could reduce its neuroprotective action against the microglia, causing it to trigger a proinflammatory M1 phenotype, with the consequent formation of interleukins, cytokines, and ROS typical of an inflammatory response, which could lead to a greater probability of developing neurodegenerative diseases. However, a correct interaction between orexins and their receptors determines an anti-inflammatory response of the microglia towards an M2 state and the production of typical secondary metabolites, providing neuroprotection. We have seen how the use of vitamin E can act directly both at the level of microglia, causing a shift from the M1 state to the M2 state, but also at the level of the orexinergic system [138–143]. Despite the few studies published to date, it is hypothesized that vitamin E is involved in the activation of the NRF2/ARE pathway, which appears to be linked to the orexin system, causing an increase in turnover and formation, thus providing greater neuroprotection”.

Error in Figure 1: In the original article, there was a mistake in Figure 1. Role of vitamin E on the orexinergic system for neuroprotection. as published. The mistake was made regarding the CD200-CD200R interaction between microglia and neurons. The corrected Figure 1 appears below.

References: With this correction, the order of some references has been adjusted accordingly. In the original article, there was an error about the CD200-CD200R interaction and the orexin nomenclature. The following original references [28,72,74,79,83–88,90,92,94–104], have been removed from the original paper as they were inherent to the link between CD200-CD200R. Original reference [28] is now updated to [2], original reference [75] is now updated to [3], original reference [76] is now updated to [4], original reference [77] is now updated to [5] and original reference [78] is now updated to [6]. References [80–82] have been changed and replaced in the paragraph regarding vitamin E as there has been an error in the references, original reference [80] is now updated to [7], original reference [81] is now updated to [8] and original reference [82] is now updated to [9].

The authors state that the scientific conclusions are unaffected. This correction was approved by the Academic Editor. The original publication has also been updated.

## Figures and Tables

**Figure 1:**
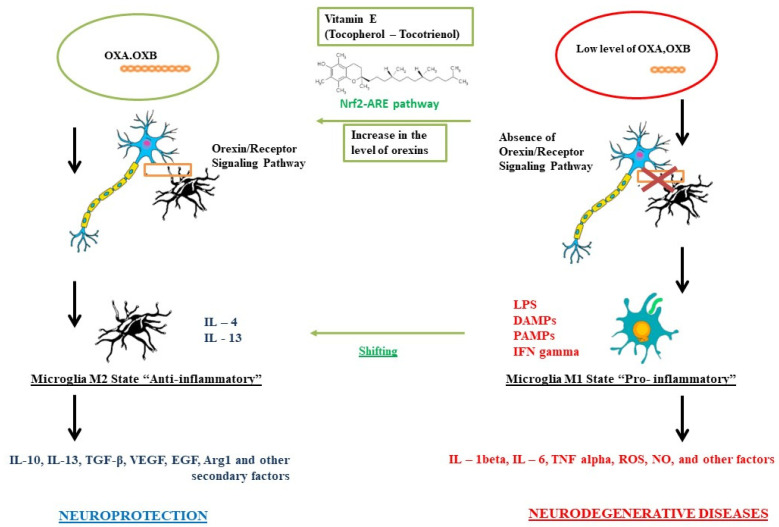
Role of vitamin E on the orexinergic system for neuroprotection.
